# Risk scores for predicting dysphagia in critically ill patients after cardiac surgery

**DOI:** 10.1186/s12871-019-0680-3

**Published:** 2019-01-10

**Authors:** Xiao-Dong Zhou, Wei-Hua Dong, Chu-Huan Zhao, Xia-Fei Feng, Wei-Wei Wen, Wen-Yi Tu, Meng-Xing Cai, Tian-Cheng Xu, Qiang-Li Xie

**Affiliations:** 1 Department of Cardiovascular Medicine, The Heart Center, The First Affiliated Hospital of Wenzhou Medical University, Wenzhou, 325000 China; 2 Department of Cardiac Care Unit, The Heart Center, The First Affiliated Hospital of Wenzhou Medical University, Wenzhou, 325000 China

**Keywords:** Dysphagia, Cardiac care unit, Prognosis, Aspiration, Pneumonia

## Abstract

**Background:**

This study aimed at developing and validating a scoring model to stratify critically ill patients after cardiac surgery based on risk for dysphagia, a common but often neglected complication.

**Methods:**

Data were prospectively collected and analyzed from January 2016 to June 2017 from 395 consecutive post cardiac surgery patients at the cardiac care unit (CCU) at a single center; 103 (26.1%) developed dysphagia. Univariate and multivariate logistic analyses were used to identify independent predictors for dysphagia. The survival nomogram was developed on the basis of a multivariable Cox model, which allowed us to obtain survival probability estimations. The predictive performance of the nomogram was verified for discrimination and calibration. Areas under receiver operating characteristic curve analysis were used to illustrate and evaluate the diagnostic performance of the novel model.

**Results:**

The final novel scoring model, named SSG-OD, consists of three independent factors: gastric intubation (OR = 1.024, 95% CI 1.015–1.033), sedative drug use duration (OR = 1.031, 95% CI 1.001–1.063) and stroke or not (OR = 6.182, 95% CI 3.028–12.617). SSG-OD identified patients at risk for dysphagia with sensitivity of 68.5% and specificity of 89.0% (OR = 0.833, 95% CI: 0.782–0.884). The positive and negative likelihood ratios were 6.22 and 0.35.

**Conclusions:**

The novel SSG-OD scoring system to risk stratify CCU patients for dysphagia is an easy-to-use bedside prognostication aid with good predictive performance and the potential to reduce aspiration incidence and accelerate recovery.

## Background

Dysphagia is an extremely frequent symptom in intensive care unit patient which may be association with the patients’ overall prognosis [1, 2]. Recent studies among patients in unselected intensive care unit (ICU) revealed 50–70% patients suffered from dysphagia [1, 3].

Similarly, critical ill patients in cardiac care unit (CCU) were thought to be at high risk of dysphagia, especially in patients who may underwent cardiac operations and required endotracheal intubation, with the prevalence of 44–87%. It has been emphasized that dysphagia is one of the significant risk factors for aspiration pneumonia and, if unrecognized, increases mortality and the length of hospitalization. Therefore, early detection of dysphagia can decrease the incidence of aspiration pneumonia [4].

Risk factors that contribute to dysphagia have been reported and included older age, congestive heart failure, sepsis, perioperative stroke, noncoronary bypass surgical procedures, transesophageal echocardiography and previous stroke. However, few studies focus on risk assessment model for categorizing patients with dysphagia risk. Grimm et al. have generated the risk of dysphagia in cardiac surgery (RODICS) score to classify patients with high risk of dysphagia who underwent more than one cardiac surgery. This score ultimately composed of 7 variables (male, body mass index (BMI) less than 20 kg/m^2^, chronic lung disease, cerebrovascular disease, ventricular assist device placement or heart transplantation, utilization of hypothermic circulatory arrest, and postoperative ventilation longer than 24 h). The 38-point RODICS score shows excellent predictive strength with the area under the receiver operating characteristics (AUROC) curve 0.75 (95% CI: 0.71–0.80).

However, RODICS score was a special to patients with endotracheal intubation who underwent cardiac operation such as coronary artery bypass, valve replacement or repair, ventricular assist device, heart transplant or combinations. However, dysphagia affects up to 16% of the critical patients, and always has been neglected in aging, cardiac insufficiency, frail and critical patients [5, 6].

Therefore, an efficacious risk assessment models for dysphagia was needed to stratify critical ill patients in cardiac care unit (CCU) for optimizing postoperative recovery and nutritional support. Our objectives were to develop a risk stratified score for critical ill patients after cardiac surgery and evaluate the diagnosis performance of this model.

## Methods

### Study design

The present study was based on an initial prospective cohort of consecutive critically ill patients at the cardiac care unit (CCU) with/without endotracheal intubation who had undergone cardiac surgery at the First Affiliated Hospital of Wenzhou Medical University (Zhejiang, China) from January 2016 to June 2017. After excluding individuals with mental disorders, aphasia, vocal cord dysfunction, oropharyngeal malignancy, Parkinson’s disease, neuromuscular diseases, neck surgery, suspicious aspiration pneumonia or refusing to participate in this study, 395 patients were enrolled. The study was approved by the ethics committee of the First Affiliated Hospital of Wenzhou Medical University Ethics Committee (Wenzhou, Zhejiang, China) and patients provided written informed consent.

Demographic and clinical data were collected in the CCU database within the hospital’s online information system. For this study, In this study, patients underwent swallowing assessment by the Gugging Swallowing Screen (GUSS) if they were: i) conscious on admission; ii) had not undergone tracheal intubation on admission; and iii) swallowing assessment were evaluated more than 4 h after tracheal endotracheal intubation. Dysphagia was defined as GUSS score of 19 points or less.

### Statistical analysis

Continuous and categorical data are expressed as mean ± standard derivations (SD) and frequencies (percentage), respectively. Student’s t-test (parametric) or the Wilcoxon rank sum test (nonparametric) was used for comparisons of continuous variables, while chi-square test was used for categorical variables. We calculated the average number of each group to be 53 by the freely available estimation software PS 3.0 (Power and Sample Size Calculation). At least 25 patients with dysphagia was were required on the basis of preliminary data. Finally, the number of participants included in our study (103 patients was diagnosed as dysphagia) rather than the number of evaluations. Univariate logistic analyses were performed to determine the unadjusted association of clinical parameters and dysphagia. Variables that were found to be different between patients with different outcomes and the parameters that were important clinically but not statistically significant all were included as candidate variables to identify independent predictors for the prognosis of dysphagia. All variables that were associated with dysphagia (*p* < 0.10) were included as candidate variables in a forward conditional stepwise logistic regression analysis to identify independent predictors for multivariate logistic regression and to calculate odds ratio (ORs) for relative risk of dysphagia. The survival nomogram was developed on the basis of a multivariable logistic model, which allowed us to obtain survival probability estimations. The predictive performance of the nomogram was verified for discrimination and calibration. In addition, the corresponding sensitivity, specificity, positive likelihood ratio, negative likelihood ratio, positive predictive value, and negative predictive value were calculated according to the area under the receiver operating curve (AUROC) results. Statistical analysis was performed using SPSS version 23.0 software (IBM, Armonk, NY) and R 3.3.1 (R Development Core Team). *P* < 0.05 was considered as statistically significant.

## Results

### Baseline characteristics of study population

Of the 395 patients enrolled in the present study, 103 (26.1%) suffered dysphagia. Mean age was 61.7 ± 12.8 years, 65.3% were men, 48.1% hypertensive and 16.5% had previous stroke. Mean left ventricle ejection fraction (LVEF) at preoperative was 56.0 ± 12.1%.

### Predictors for dysphagia

As shown in Table 1, patients with dysphagia had higher incidence of previous stroke (35.9% vs. 9.6%), longer endotracheal intubation (24 (15–38) vs. 19 (0–24) h) and sedative drug use duration (9 (6–17) vs. 6 (0–10) h). Univariate logistic regression analysis identified endotracheal intubation duration (*p* < 0.001; OR = 1.027, 95% CI 1.015–1.038), gastric intubation duration (*p* < 0.001; OR = 1.030, 95% CI 1.021–1.038), sedative drug use duration (*p* < 0.001; OR = 1.028, 95% CI 1.013–1.042) and stroke (*p* < 0.001; OR = 5.286, 95% CI 3.019–9.255) as possible predictors of dysphagia (Table 2).

**Table 1:** Characteristics of study population

**Variable**	All	Non-DG	DG	*P*
*N*	395	292	103	
Age, year	61.7 ± 12.8	61.2 ± 12.8	63.3 ± 12.8	0.198
Male, n (%)	258 (65.3%)	188 (64.4%)	70 (68.0%)	0.549
Height, cm	163.7 ± 7.3	163.4 ± 7.1	164.8 ± 7.6	0.201
Weight, kg	62.4 ± 11.1	62.2 ± 11.1	63.3 ± 11.2	0.441
BMI, kg/m^2^	23.4 ± 3.3	23.4 ± 3.3	23.4 ± 3.2	0.948
Hypertension, n (%)	190 (48.1%)	142 (48.6%)	48 (46.6%)	0.732
Diabetes, n (%)	99 (25.1%)	77 (26.4%)	22 (21.4%)	0.356
Previous stroke, n (%)	65 (16.5%)	28 (9.6%)	37 (35.9%)	< 0.001
Atrial fibrillation, n (%)	61 (15.4%)	41 (14.0%)	20 (19.4%)	0.206
CABG procedure, n (%)	91 (23.0%)	65 (22.3%)	26 (25.2%)	0.586
Echocardiography parameters				
LVEF (%)	56.0 ± 12.1	56.0 ± 11.9	55.9 ± 12.5	0.693
LAD (mm)	42.9 ± 8.3	42.7 ± 8.6	43.5 ± 7.5	0.503
LVDD (mm)	51.6 ± 8.1	51.8 ± 8.4	51.2 ± 7.2	0.192
Endotracheal intubation, hours	21 (0–25)	19 (0–24)	24 (15–38)	< 0.001
Gastric intubation, hours	0 (0–22)	0 (0–19)	24 (0–91)	< 0.001
Sedative drug use duration, hours	6 (0–13)	6 (0–10)	9 (6–17)	0.004

Note: *CABG*: coronary artery bypas**s** grafting; *DG*: dysphagia; *LVD*: left atrial diameter; *LVDD*: left ventricular end-diastolic diameter; *LVEF*: left ventricular ejection fraction;

**Table 2:** Univariate analysis

	OR	95% CI	*P*
Age	1.012	0.994–1.03	0.198
Male	1.173	0.727–1.893	0.512
BMI	1.003	0.919–1.095	0.948
Hypertension	0.922	0.588–1.446	0.723
Diabetes	0.758	0.443–1.299	0.314
Previous stroke	5.286	3.019–9.255	< 0.001
Atrial fibrillation	1.475	0.818–2.66	0.196
LVEF	1.000	0.981–1.018	0.958
CABG procedure	1.179	0.699–1.99	0.537
Endotracheal intubation	1.027	1.015–1.038	< 0.001
Gastric intubation	1.030	1.021–1.038	< 0.001
Sedative drug use duration	1.028	1.013–1.042	< 0.001

Note: *CABG*: coronary artery bypas**s** grafting; *LVEF*: left ventricular ejection fraction;

### Development of the risk score for predicting dysphagia

**Fig. 1:**
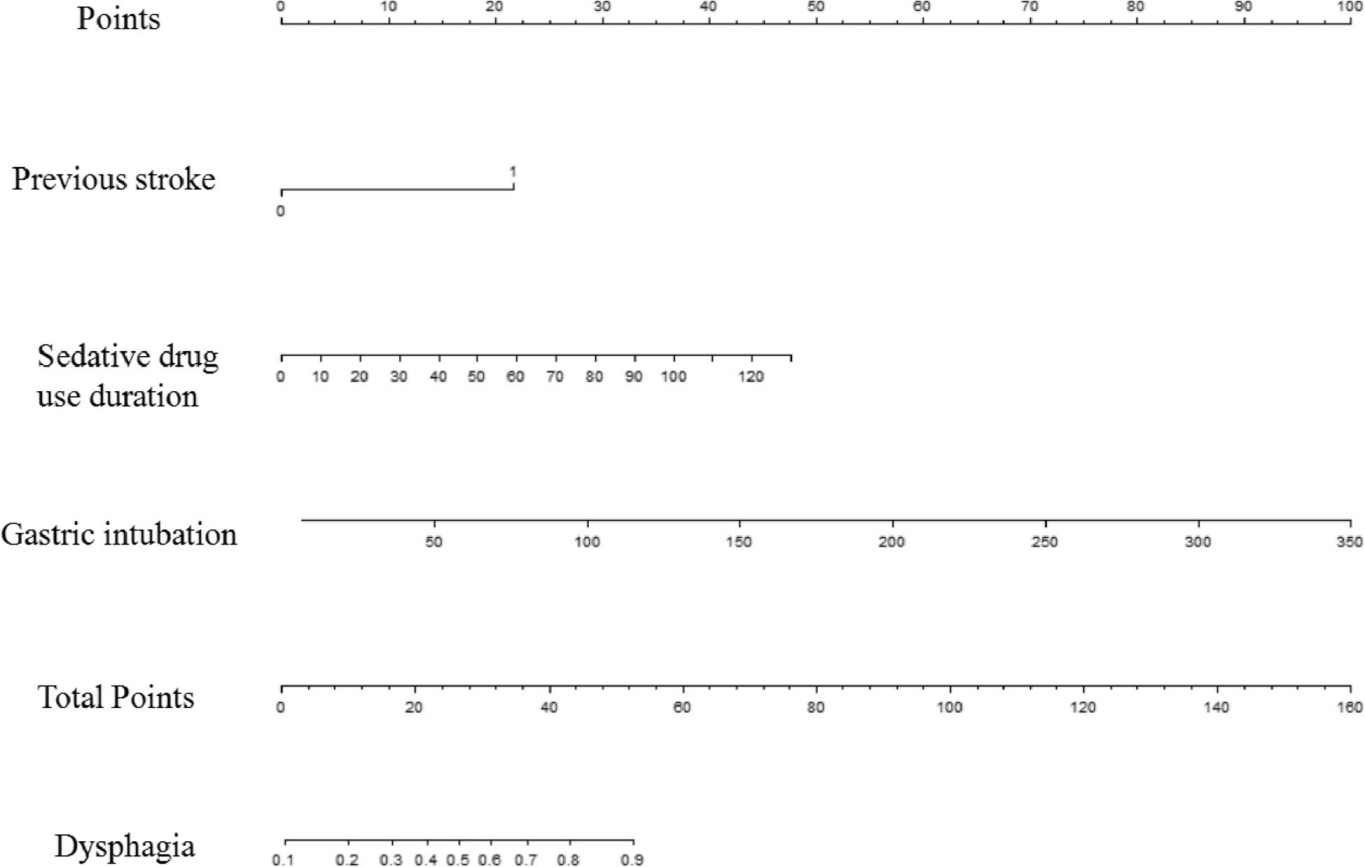
The nomogram of SSG-OD

In multiple logistic analyses, Endotracheal duration (OR = 0.971, 95% CI 0.940–1.012), gastric intubation (OR = 1.048, 95% CI 1.029–1.066), sedative drug use duration (OR = 1.035, 95% CI 1.004–1.067) and stroke (OR = 7.057, 95% CI 3.378–14.742) were independent risk factors for dysphagia (Table 3). Endotracheal intubation was not significantly correlated with dysphagia and then was unselected. The final model included gastric intubation (OR = 1.024, 95% CI 1.015–1.033), sedative drug use duration (OR = 1.031, 95% CI 1.001–1.063) and stroke or not (OR = 6.182, 95% CI 3.028–12.617). Finally, Stroke- sedative drug use duration- gastric intubation for dysphagia (SSG-OD) was established through a prognostic nomogram (Fig. 1). To use the nomogram, the first variable was located. A straightline was then drawn upwards to the point’s axis to determine the points received for the variable. The process was repeated for the other variables and the points were then summated for each variable. The sum of these numbers was located on the total points axis and a line was drawn downward to the survival axes to determine the likelihood of dysphagia.

**Table 3:** Multivariate analysis

	OR	AOR
Previous stroke	7.057 (3.378–14.742)	6.182 (3.028–12.617)
Endotracheal intubation	0.971 (0.940–1.012)	
Gastric intubation	1.048 (1.029–1.066)	1.024 (1.015–1.033)
Sedative drug use duration	1.035 (1.004–1.067)	1.031 (1.001–1.063)

Note: *OR* = odds ratio; *AOR* = adjusted odds ratio

### Prognosis performance

**Fig. 2:**
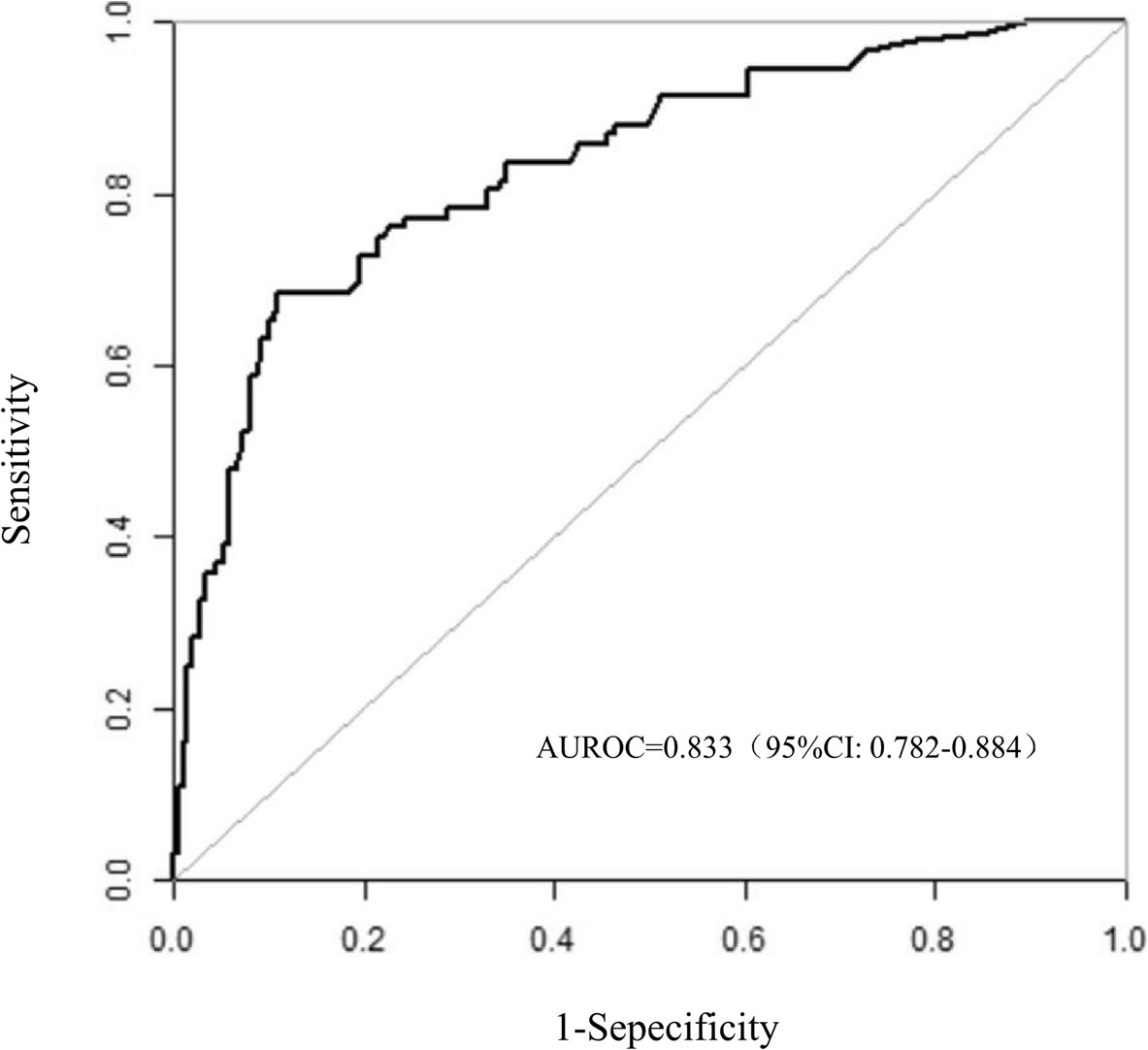
ROC analysis of the prognostic efficiency of SSG-OD

In performance analysis (Fig. 2), the AUROC of SSG-OD was 0.833 (95% CI: 0.782–0.884). When applying the optimal cutoff value for predicting dysphagia, sensitivity and specificity were 68.48 and 89.00%, respectively. Moreover, the positive and negative likelihood ratios were 6.22 and 0.35, and the positive and negative predictive values were 0.733 and 0.865, respectively. As part of the assessment of its discriminator power, the new SSG-OD scoring system was compared to the RODICS score (Table 4). In the present study, although RODICS score was a reliable model to predict dysphagia in critically ill patients after cardiac surgery (sensitivity, 0.712; 95% CI: 0.697–0.728), SSG-OD score showed a better prognosis performance. In the stratified analysis, SSG-OD score had a significantly higher predictive ability in critically ill patients with endotracheal intubation after cardiac surgery (all *P* < 0.001).

**Table 4:** Diagnostic accuracy

	SAGE-OD	RODICS
AUROC	0.833	0.681
Cut-off point	1.5	5.5
Sensitivity	68.5%	68.0%
Specificity	89.0%	62.4%
Youden index	0.575	0.304

Note: *AUROC*: area under the receiver operating characteristic curve

## Discussion

SSG-OD score is an easy-to-use bedside scoring model for predicting dysphagia in critical ill patients after cardiac operation, but also has excellent prognosis accuracy as comparison of the existing RODICS score, which might have substantial value in reducing the incidence of aspiration and pneumonia.

Patients with stroke have a reported frequency of dysphagia in recent studies, ranging from approximately 50–80% [7]. Mechanics of swallowing dysfunction included reduced hyoid excursion, reduced tongue base retraction, and reduced pharyngeal shortening [8–10]. Many studies have found that dysphagia is association with subtype and severity of stroke [11]. Sedation may cause damage on pharyngeal function procedure, pharyngeal muscle contraction, potentially increasing the risk for pulmonary aspirations [12]. Sedation has inhibitory effects on swallowing reflexes increasing latency to initiate swallowing and decreasing spontaneous swallow frequency [13, 14]. Sedation has an effect on pharyngeal muscle contraction duration and velocity and causes a delayed increase on esophageal sphincter pressure [15]. Furthermore, sedatives may result in delay in the improvement of mental status, which in turn may prolong duration of endotracheal intubation and nasogastric tube [16].

In our study, the results demonstrated that the duration of gastric intubation (nasogastric tube), as a novel variable, have certain prognosis value for predicting dysphagia. It is a seemingly simple procedure but prolong gastric intubation can sometimes result in dysphagia. Nasogastric tube as a risk factor for dysphagia and aspiration pneumonia, with an incidence of pneumonia of 38–44% [17–19]. Nasogastric tube disorganized the pharyngeal contraction, narrow upper esophageal sphincter and then lead to slower swallowing velocity [20]. Prolonged placement of the nasogastric tube appears to induce acidic gastroesophageal reflux and therefore contribute to pharyngeal mucosa damage [21].

Prolonged endotracheal intubation already been identified as a well-recognized risk factor of dysphagia in patients after cardiac surgery [4, 22]. Earlier reported studies focusing on specific intubation durations: > 24 h, > 48 h and from 8 to 28 h [4, 23, 24]. Skoretz et al. classified endotracheal intubation into four groups: < 12 h, > 12 to ≤24 h, > 24 to ≤48 h, and > 48 h [22]. The findings demonstrated that patients with prolong endotracheal intubation required a almost twofold increase in risk of dysphagia for every additional 12 h. RODICS score also defined postoperative ventilation longer than 24 h as 1 score [25]. Similarly, prolong endotracheal intubation was also defined as > 24 h in the SSG-OD score. In this study, univariate analysis reported that endotracheal intubation is significantly associated with dysphagia. However, when adjustment ware made for gastric intubation duration, endotracheal intubation were not significantly correlated with dysphagia.

A few potential limitations need consideration. First, because our study population came exclusively from a single center, potential selection bias might exist. Second, in the absence of a comparative large-scale population, the performance analysis of our final predictive model had to be completed on the same database. Multi-center large-scale studies are needed to further verify its applicability of SSG-OD. Third, although criterion for dysphagia is an instrumental assessment using videofluoroscopy.

(VFSS) [26, 27], the diagnosis was based on GUSS with 100% sensitivity and 69% specificity to predict aspiration risk.

## Conclusion

In summary, we derived the first scoring model (SSG-OD) that was specific to critically ill patients after cardiac surgery to stratify the mortality risk of dysphagia. Moreover, SSG-OD shows an improvement of discriminative accuracy compared with existing score RODICS score.

## Abbreviations

AUROC: area under the receiver operating curve
BMI: body mass index
CCU: cardiac care unit
GUSS: Gugging Swallowing Screen
ICU: intensive care unit
LVEF: left ventricle ejection fraction
OR: odds ratio
RODICS: score the risk of dysphagia in cardiac surgery score
SD: standard derivations
VFSS: videofluoroscopy
